# Walls offer potential to improve urban biodiversity

**DOI:** 10.1038/s41598-020-66527-3

**Published:** 2020-06-18

**Authors:** Chundi Chen, Longfei Mao, Yonggui Qiu, Jian Cui, Yuncai Wang

**Affiliations:** 1 Joint Laboratory of Ecological Urban Design (Research Centre for Land Ecological Planning, Design and Environmental Effects; International Joint Research Centre of Urban-Rural Ecological Planning and Design), College of Architecture and Urban Planning, Tongji University, Shanghai, China; 2 Bioinformatics Center, College of Biology, Hunan University, Changsha, Hunan China; 3 Institute of Botany, Jiangsu Province and Chinese Academy of Sciences, Nanjing, China

**Keywords:** Ecology, Biodiversity, Urban ecology

## Abstract

Within urban environment with high-rise buildings and structures, walls represent the most common vertical spaces. Conventionally, such spaces are viewed as abiotic areas, where spontaneous flora is neglected. Through investigations in a typical mountainous city Chongqing, this study concerns the spontaneous species diversity on walls and the influences of wall factors and the adjacent environment. A total of 239 vascular plant species belonging to 172 genera and 75 families were found; 90% of the species are indigenous. More fern species inhabit walls, compared with xerophytes that dominate general urban environment. Variation partitioning indicated that wall attributes played a more important role in explaining the total variation in wall vegetation composition and structure than did the adjacent environment. Given that there are limited possibilities to extend more green space in urban land environment, we support a “let Nature take its course” approach to improve urban biodiversity, where vertical urban space with spontaneous flora can act as a valuable complement to biodiversity and ecosystem services in dense urban environment. This study should raise urban designers’ and ecosystem managers’ awareness of the possibilities of this type of informal, unconventional habitat as a “supplement” for urban greening and landscaping.

## Introduction

Globally, continuing urbanisation is projected to reach 66% by 2050 and over 6 billion people are expected to live in urban environment^1^. As the most highly disturbed system, urban ecosystems experience 70–90% of human activity^2^, even though current urban areas cover only 2–4% of the world’s surface^3,4^. Within such small portions of the earth’s surface, land resources are limited and conflicts between various land uses often occur, while materials and energy consumption are intensified with unprecedented impacts on environment. Under current circumstances, there is either not much land to add new urban green space or it is not feasible to transform already-concreted land to natural habitats.

However, we have long neglected the fact that a city is a three-dimensional space that has a large quantity and variety of walls. They may offer great spatial potential for colonisation by naturally dispersed vegetation, especially for highly dense cities with notably limited land resources. Generally, such concrete space is not conventionally regarded as a growing substrate. Instead, it is used as a “container” to carry soil and nutrients to grow plants. Recent observations and studies have started to notice that these vertical niches can nurse uncultivated, spontaneous plants^5^, although such “habitats” may be informal, precarious and transitory. Spontaneous vegetation is a cosmopolitan mix of species that grow and reproduce without human intent^6^. They are highly adaptable to nutrient deficiency and an alkaline substrate, and may self-sustain and self-assemble without human maintenance and management. Different from two-dimensional land environment with deeper soil and good hydrological conditions, the species composition and structure seems to depend more on extrinsic conditions, such as the surrounding environment, and intrinsic attributes, such as the wall material^7^.

Although these silently growing species are often neglected, they can contribute important ecosystem services in the context of the combined impact of urbanisation and climate change^6^. They promote urban plant diversity and provide stopover opportunities for insects and birds in urban environment full of decorative and/or ceremonial species^8^. As a part of the architecture, walls with species are also well known to increase building energy efficiency and provide a cooler microclimate^9^.

Though studies on spontaneous wall plants have long been conducted mainly in European cities in the temperate climatic zone^5^, only recently, such unusual niches have started attracting attention in China and a few empirical studies have been carried out in Hong Kong^10–12^, Nanjing city^13^ and Xi’an city^14^. However, most studies focus on ancient walls such as old church walls in Europe^5,15^ and the Nanjing city stone wall of the Ming Dynasty^13^. These older walls are often made from stones that substantially mirror natural cliff and rocks, so wall species are dominated by chasmophytes, as in the “Urban Cliff Hypothesis”^16,17^. It speculates that a large portion of the species that spontaneously colonised cities were originally from rock habitats, such as cliff and stones, because the urban built environment (e.g. pavements, walls and roofs) resemble such habitats^16,17^. For example, European chasmophytes *Brassica cretica* subsp. *cretica*, *Centaurea raphanina* subsp. *mixta*, *Erysimum corinthium* and *Silene spinescens* found on the walls of a castle were also found commonly on adjacent rocky slopes^18^.

However, walls in modern cities are of varied construction materials, such as brick, concrete, mortar and tiles, which have significantly changed the wall environment and attributes. More attempts are needed to deal with different geographical areas and more types of ordinary wall in today’s cities to provide empirical information about varied urban habitats.

As the largest landlocked city in Southwest China, Chongqing is a typical “mountain city” built among hills. The complex terrain and high-rise buildings generate a large number of walls, including building walls, boundary walls and retaining walls used for reinforcing hill slopes^5^. Additionally, the city is located in a region with great biogeographical diversity at both national and global scales. This study therefore investigates spontaneous vegetation composition and structure on walls in Chongqing City to assess if it supports the urban cliff hypothesis that influences the patterns of urban biodiversity. This is an additional attempt to understand the impact of wall environment in the different biogeographical regions of China. The findings from this humid-tropical study can provide complementary comparative data and further facilitate the understanding of a unique component of urban niches. This should raise urban designers’ and ecosystem managers’ awareness in selecting alternative plant species for more ecosystem services in cities, and inform them of the possibilities of this type of informal environment as a “supplement” to urban greening and landscaping.

## Methodology

### Study area

**Figure 1:**
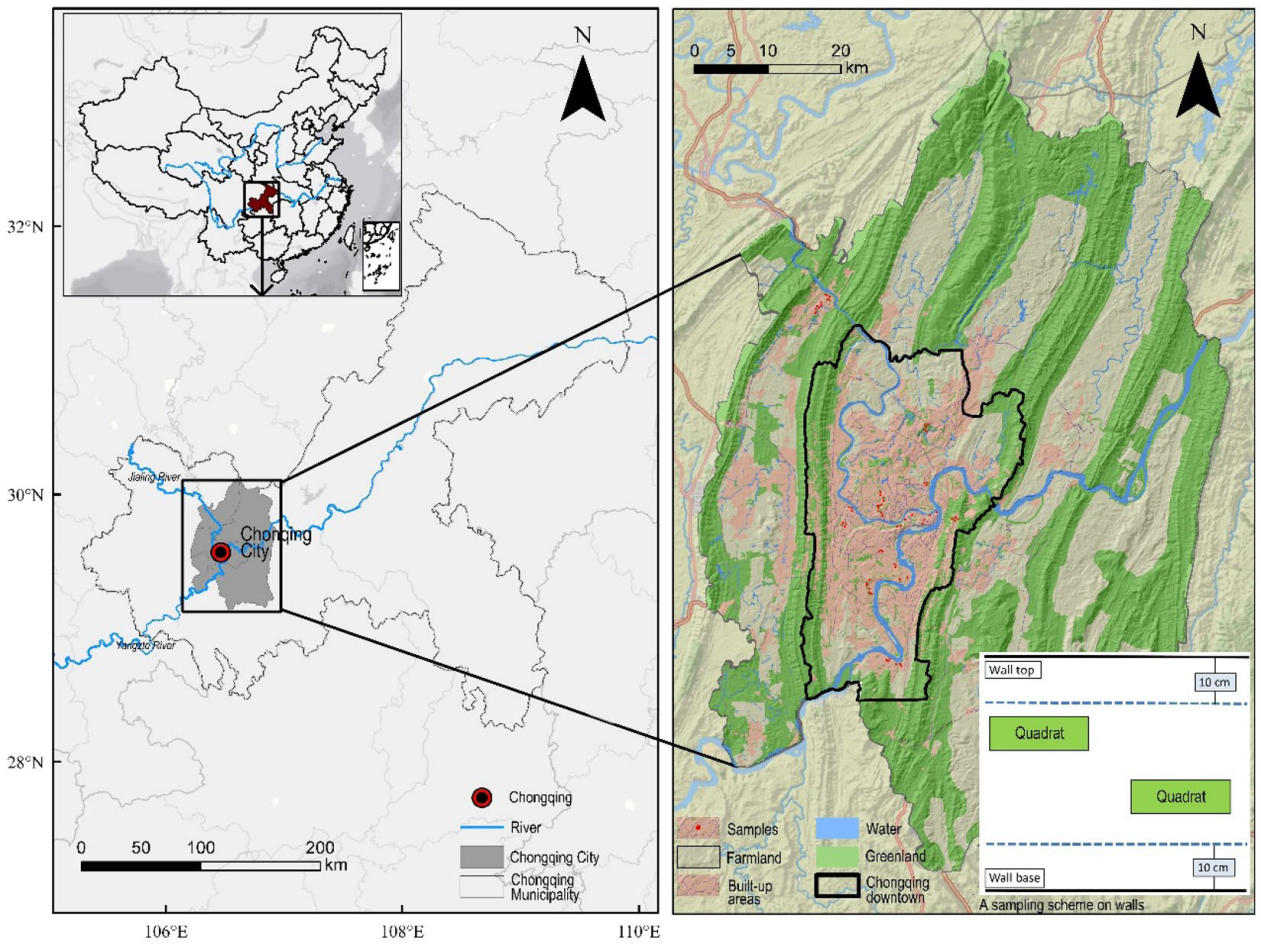
The study location area and a sampling scheme (the right basemap obtained from ArcGIS Online owned by National Geographic, Esri, DeLorme, HERE, UNEP-WCMC, USGS, NASA, ESA, METI, NRCAN, GEBCO, NOAA, iPC. http://www.arcgis.com/home/item.html?id=b9b1b422198944fbbd5250b3241691b6).

Chongqing City (105°11′ E-110°11′ E, 28°10′ N-32°13′ N) is located at the confluence of two rivers, the Jialing and Yangzte Rivers (Fig. 1). It has nine districts, covering an area of 1329km^2^ with over 8 million urban residents. Around three-quarters of the municipal land is mountainous. The region is characterised by a northern subtropical humid monsoon climate with an average annual precipitation of 1129 mm, annual average temperature of 15–18 °C^19^. There are fewer than 20 frost days per year and only seldom snow above 1000 m above sea level (a.s.l.) on the mountains^19^. The flora and birds in the municipality account for 21% and 29% of all seed plants and birds in China, respectively, and include a number of ancient, endemic and rare species^20^. Subtropical broad-leaved evergreen forest is the regional climax vegetation. The main species are *Ficus virens*, *Ficus beipeiensis*, *Pterocarya stenoptera* and *Cinnamomum camphora*^19^.

### Vegetation survey

Based on the Chongqing City land use map^21^ (with a resolution of 30 m), we reclassified land use into three types: parks, concrete environment (residential, roads, plazas, etc.) and abandoned areas. Then we used ArcGIS 10.3 software “Create Random Points” tool^22^ to generate 15 randomly-placed points within the boundaries of three land use types and nine districts of Chongqing City. These points with GPS information can be located on the ground and used as sampling sites. A survey was conducted during September and October, 2017 and 2018. Generally, there is a relatively low incidence of plants growing on walls in cities^23^. To avoid “empty” samples, only vegetated walls were considered for this study. Additionally, at least one of each wall type (building, boundary and retaining walls) was included. After excluding some sites that were either inaccessible or did not meet the requirements above, 4–6 sites (each site with one wall) in each of three land use types in nine districts were finally sampled.

All sampled walls were geographically positioned within 1 m accuracy using a GPS recorder. On each wall face, we laid 2–4 rectangular quadrats (3 × 1 m with the short axis vertical), excluding 10 cm buffers at the top and bottom to avoid possible edge effects (Fig. 1). Similarly, to avoid “empty” samples, a quadrat must be positioned in a plot with at least two species. A total of 413 quadrats on 136 walls were sampled. We recorded all plant species names and coverage, and calculated species abundance and richness for every quadrat and every sampled wall (the average of all quadrats on a wall). The species information was assessed by one botanist to attain consistency. The nomenclature and species attributes, including origin, follow the Flora of China (http://frps.eflora.cn/), the local Sichuan Flora^24^ and Chongqing Vascular Plants^25^. Exotic plants were defined as taxa whose presence was because of intentional or unintentional human agency^26^; crop plants or archaeophytes (i.e. introduced before 1500 A.D.) were excluded because of controversy over their status. We did not subdivide indigenous species into different regional origins within China. Each variety and subspecies was treated as a full species.

### Wall environment survey

The attributes of the walls and their adjacent influences were recorded. Partially based on Hong Kong’s stone wall study^11^, wall attributes in the study comprise three subgroups: wall intrinsic attributes, wall extrinsic attributes and wall joint attributes. Adjacent influences include two subgroups: human management and land use type. Table 1 lists the detailed indicators and their main ecological implications. Most indicators can be measured quantitatively. For categorical variables, such as degree of wall degradation and roughness, a 5-point Likert scale from “lowest” (1) to “highest” (5) was used.

**Table 1:** A summary of possible wall influences and variables.

Influence		Indicator	Main ecological implication	Influence level
Wall Attributes	Wall intrinsic attributes	Height, *length*, slope, color, *aspect*, *wall type*, wall material, wall degradation degree, *wall roughness*	Directly relevant to plant growing substrate	Community level
	Wall extrinsic attributes	Humidity, wall shade, vine coverage, *moss/lichen coverage*		
	Wall joint attributes	Joint material, joint density, *joint degradation degree*, joint size		
Adjacent influences	Management	Human disturbance degree (e.g., how often cleared), management level of surrounding environment	Directly relevant to impacts of human activity	Landscape level
	Land use type	land use type surrounding the sampled walls		

Note: Italic means non-significant variables and were deleted for analysis.

### Statistical analysis and modelling

Analyses were conducted at the wall patch level (a whole sampling wall as the minimum analysis unit). Species coverage data were standardised by Hellinger transformation^27^ and used as the response variable. Environmental data include 19 explanatory variables that were log10 (X + 1)-transformed before analysis^28^. The species-wall relationship was calculated by constrained gradient analysis methods commonly used in quantitative ecology^29^. Through detrended correspondence analysis (DCA), the length of the first DCA axis was greater than 6 indicating that canonical correspondence analysis (CCA) was appropriate^29^. The correlations of each variable to the two most explained gradients of CCA ordination were used to indicate the importance of these variables to wall vegetation composition and structure. Forward selection procedures were used for an initial screening of the candidate indicators (P ≤ 0.05).

Then the function “varpart” was performed to calculate the independent and shared effect of variable groups in the “vegan” package of R 3.1.3^30^. This variation partitioning approach uses redundancy analysis ordination (RDA) and extracts four variation fractions: the independent effects of wall attributes and adjacent influences, their shared effects and the unexplained. It allows comparisons of the relative importance of different influences. Collinear variables are not required to be removed before partitioning in this method^30^. Monte Carlo permutation tests with 999 permutations were developed to evaluate the significance of variables separately^31^. Analysis results with P ≤ 0.05 are considered statistically significant.

## Results

### Plant species composition on walls

**Figure 2:**
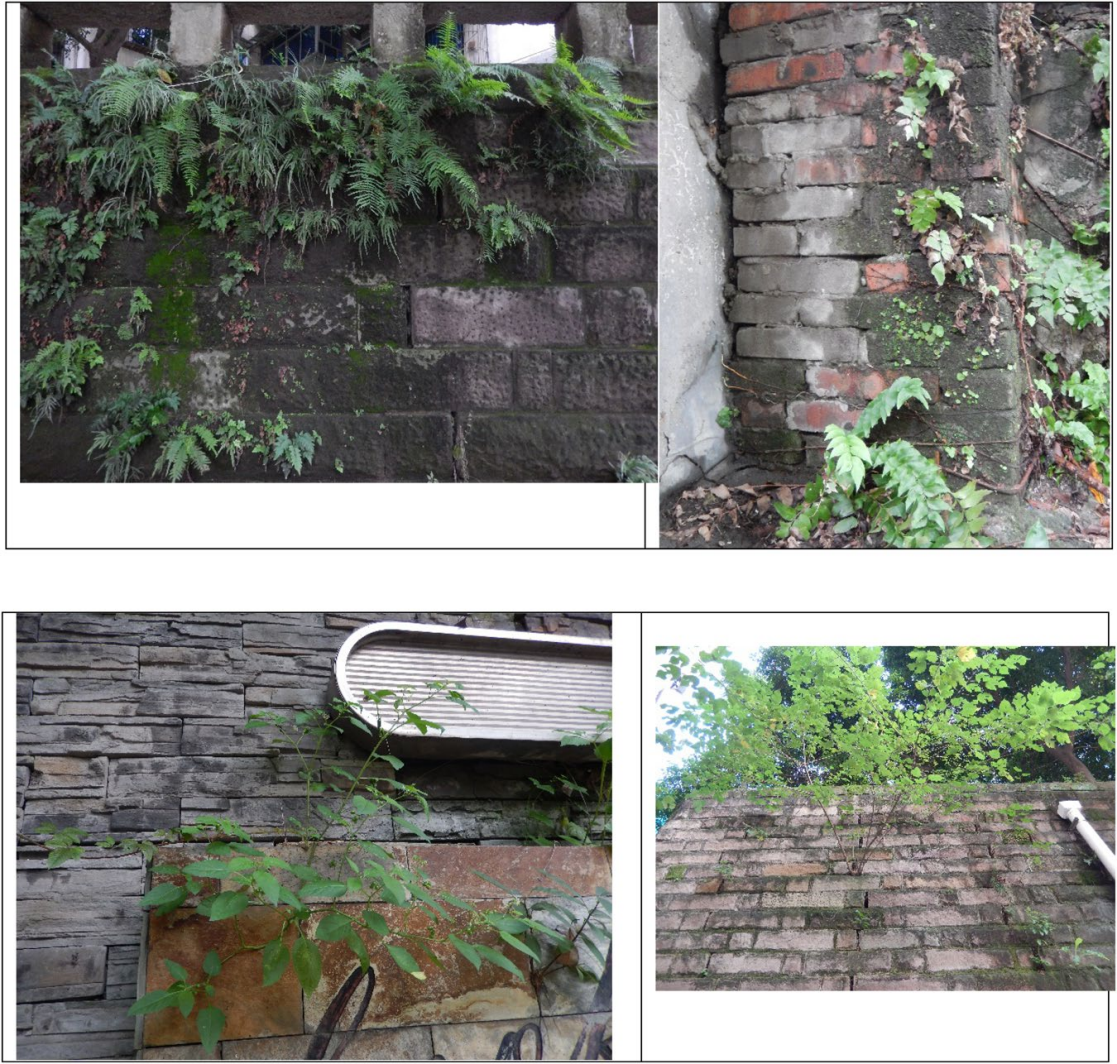
Spontaneous plants on walls in Chongqing city (Photographed by Chundi Chen).

A total of 239 vascular plant species belonging to 172 genera and 75 families were found across the 413 quadrats. The three dominant families were Compositae (12.4% of total), Gramineae (5.2%) and Urticaceae (5.2%). The most frequent species were *Pteris vittata* (78.3% of all quadrats), *Pteris multifidi* (49.3%), seedlings of *Broussonetia papyrifera* (45.4%), *Cyrtomium fortune* (42.1%), seedlings of *Ficus virens* (41.5%), *Youngia japonica* (30.4%), *Cyclosorus parasiticus* (22%), *Oxalis corniculata* (19.5%), and *Rorippa indica* (17.3%). They greatly support natural greening of urban vertical space (Fig. 2, species list see Appendix A).

Species richness per quadrat ranged from 1 to 29, with an average of 7.13 (SD = 4.45). The overall species richness per sampled wall ranged from 3 to 39 with an average of 13.5 (SD = 7.72). Within the three land use types, “parks” had the highest species richness followed by abandoned areas and a concrete environment (one-way ANOVA sig=0.003, see Appendix B). This result is not surprising and may suggest that adjacent influences where walls located are likely indicators of spontaneous flora occurrence.

We found a diverse range of plant life forms on walls, from woody to fern and vine (Table 2). Obviously, the dominant species are perennial herbs (34.7%) and annual herbs comprise. In an unanticipated result, woody species (seedlings of trees and shrubs) also accounted for 20.8%. 11.7% fern and 6.7% vine species inhabit walls. More fern and vine species indicate more moisture and suggest some uniqueness of the walls as habitats.

**Table 2:** Life forms of spontaneous vegetation on walls.

Percentage/%	Life forms	No. of species
25.9	Annual/biennial herbs	62
34.7	Perennial herbs	83
7.9	Shrubs	19
12.9	Trees	31
11.7	Fern	28
6.7	Vine	16
100	Total	239

Of the 239 species, one species had no clear record of origin; 215 were indigenous and 23 were exotic species of which nine were identified as invasive. The most common exotic species were *Conyza canadensis*, *Erigeron annuus* and *Oxalis corymbosa*, which occurred in 8.7%, 3.5% and 2.3% of all quadrats, respectively. Individual exotic species’ coverage varied markedly between quadrats, ranging from 0 to 40%. The exotic species richness varied by quadrats ranging from 0 to 3, with an average of 0.3 (SD = 0.59). Overall, the very small proportion of exotic species suggests that the area is unlikely to be subject to invasion risk. Additionally, some species, such as *Alternanthera philoxeroides* and *Ageratina adenophora*, reported as strong invaders in land environment in China; but occurred only rarely on the walls throughout the survey (0.8% and 1% of all quadrats, respectively).

### Wall attributes

We surveyed 136 walls in downtown Chongqing City. Most were in Yuzhong, Shapingba and Ba’nan, the old districts. Retaining walls were most common, accounting for 78% of all surveyed walls. The rest are building walls and boundary walls, 11% each. Two thirds of the walls were piled up stones, while 28% were bricks and concrete walls were only 6%. A large proportion (45%) of walls were up to 2 m high, while 23% of the walls were 3–4 m, mainly being building walls and retaining walls. Only 2.5% of the walls had an extreme moisture status with noticeable water seepage on the wall facade. Generally, such walls were beside water sources or had high shade coverage from the surroundings.

Concrete walls generally had no bonding material; but the brick and stone walls often (73%) had cement lime mortar as adhesive underneath and between bricks and tiles. Recent walls often used cement mortar (around 11.7%). Only a small number of masonry walls in exposed sites were without bonding. During the survey, greening workers and/or real estate managers said that they had no rules about regular wall clearance. Overall, various old and new buildings co-exist within the downtown and the wall characteristics and properties varied widely. This implies that the walls may provide diverse living conditions for plants, such as hygrophilous or drought-tolerant plants.

### Correlation between the variables and wall plants

**Figure 3:**
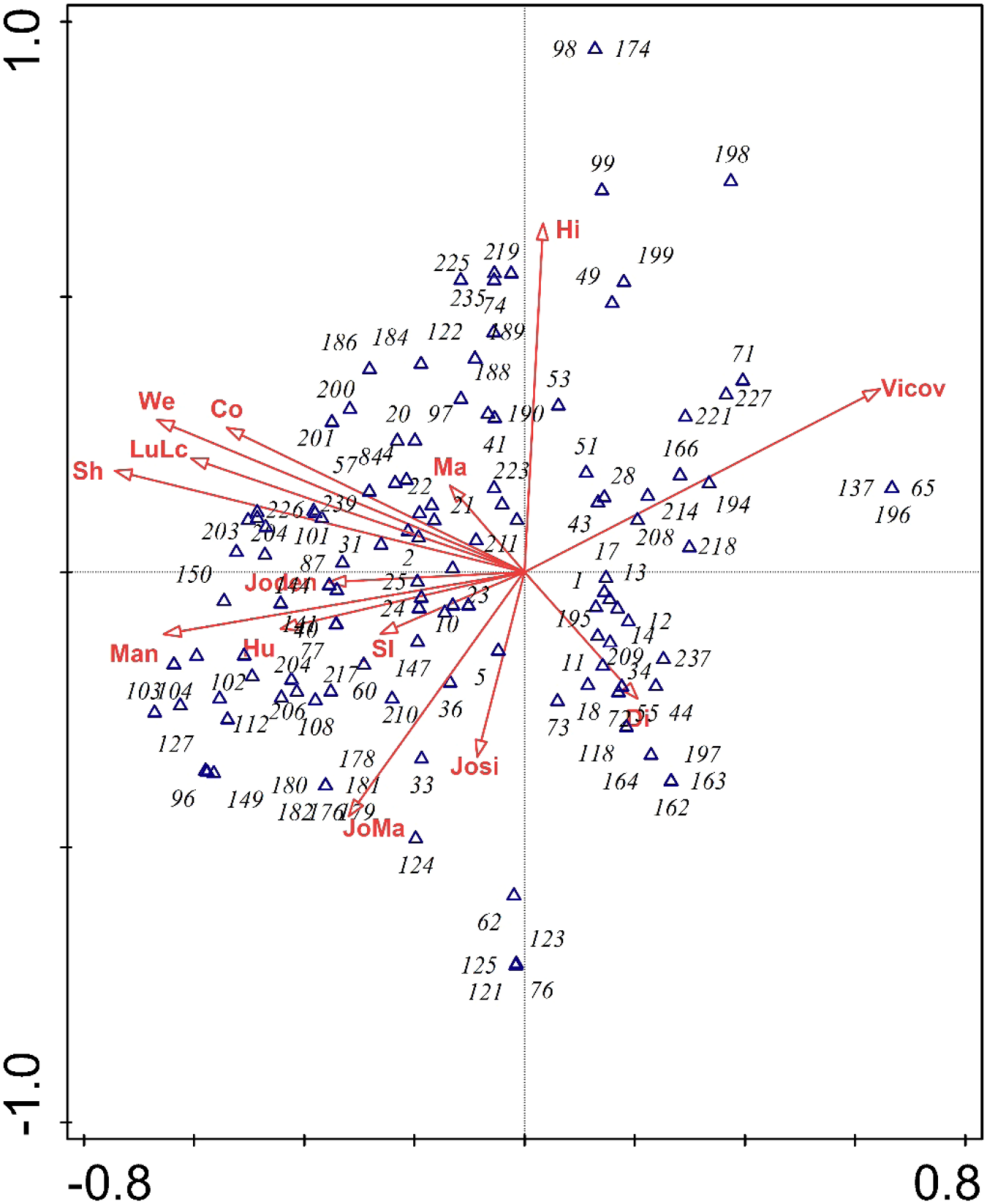
Canonical Correspondence Analysis (CCA) for spontaneous vegetation on walls of Chongqing City (Sl: slope; Co: color; Hi: height; Ma: material; JoMa: Joint material; Josi: Joint size; Hu: Humidity; Vicov: vine coverage; Sh: shade; We: wall degradation degree; Man: management level of surrounding environment; Di: Human disturbance degree; Joden: joint density; LuLc: land use type surrounding the sampled walls.

For all species at the community level, forward selection suggested 13 significant variables among the 19 variables of the wall attributes for further analysis (Fig. 3). Vine coverage and wall shade have the strongest correlation with the first axis in CCA. These factors have certain correlations among each other and all are (in)directly relevant to wall humidity. This indicates that moisture plays a decisive role in structuring the vegetation on urban walls. Along the negative direction of the first axis were concentrated ferns, seedlings of woody species, hygrophilous plants and moisture-tolerant plants (e.g., *Oplismenus undulatifolius* as no. 31 in Fig. 3). In contrast, saplings that do not need as much shade and moisture as sprouts generally dispersed along the positive direction (e.g. *Ficus virens* as no. 194 in Fig. 3). This supports the idea that the first axis is closely related to water-associated factors. The vertical axis has high correlations with joint size, wall height and joint material. Some woody species, like *Sambucus williamsii* (no. 10 in Fig. 3) and *Citrus maxima* (no. 147 in Fig. 3) tend to grow in walls with larger wall joints. This indicates that wall joints are crucial to the composition and structure of wall vegetation. Together wall attributes and adjacent influences explain 28.3% of the variance of the vegetation.

Within the management subgroup, Man (management level of surrounding environment) and LuLc (land use type surrounding the sampled walls) have strong correlations with the negative direction of the first axis. These two variables actually correlate each other, i.e., the land use of parks has intensive management whereas abandoned areas seldom have human management. Unsurprisingly, some common greening and landscaping species, like *Microlepia strigosa* (no. 87 in Fig. 3), *Pteris multifidi* (no. 57 in Fig. 3) and *Chlorophytum comosum* (no. 201 in Fig. 3) are likely to disperse along the variables Man and LuLc.

Overall, vine coverage, wall shade and wall height were the most important factors among all variables in influencing vegetation composition and distribution, accounting for 5.2%, 4.8% and 4.5% of the variance in vegetation (see Appendix B).

### Relative effects of wall attributes and adjacent influences

**Figure 4:**
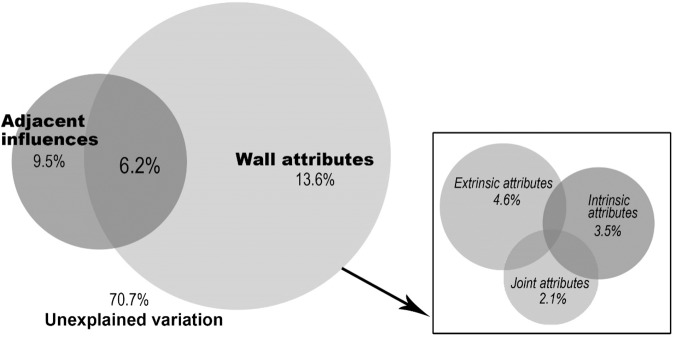
Variation partitioning used to determine the effects of wall attributes and adjacent influence and their interactions on spontaneous species. Left: variation of the species data matrix is explained by two explanatory groups (wall attributes and adjacent influences). Right: wall attributes variation is explained by the three subgroups of wall intrinsic attributes, wall extrinsic attributes and wall joint attributes. Each circle corresponds to a group of explanatory variables. Numbers within the non-overlapping parts of circles are the proportion of variation explained by each group of variables alone. Numbers within overlapping parts represents the joint explanation.

The total variation in wall vegetation composition and structure captured by all selected variables was 29.3%; the unexplained part is 70.7% (Fig. 4). Decomposition of the variation showed that the largest fraction of the variance was accounted for by wall attributes (13.6%), followed by the adjacent influences (9.5%). The joint effect of two groups made the smallest contribution with explanatory power of 6.2%. Considering the variable group of the wall attributes separately, variation partition analysis indicated that the extrinsic attributes explained most of the variance (4.6%), followed by the intrinsic attributes (3.5%). The subgroup of wall joint attributes showed a lesser independent effect (2.1%). Their joint effects are so small that they did not pass tests of significance (see Appendix C). This agrees with the analyses of individual variables above.

## Discussion

### Spontaneous vegetation on walls

In general, in urban two-dimensional land environment, spontaneous plants total 129 species in 101 genera from 48 families in Ningbo City^32^, 107 species in 89 genera from 32 families in Shanghai downtown^33^, 95 species in 75 genera from 37 families in Xi’an City^14^, and 128 species in 98 genera from 32 families in Beijing Olympic Forest Park^34^. In our study, walls with comparatively harsher conditions, supported 239 vascular plant species belonging to 172 genera and 75 families. Similarly, a study of the extant ancient city wall of Nanjing City found 159 species in 125 genera of 70 families^13^. A recent study of modern urban walls (stone, concrete or brick) in Christchurch and Dunedin cities, New Zealand, found 117 vascular and non-vascular species in 41 families, and 70 species in 26 families respectively^23^. It seems that wall habitats perform well compared with general urban environment. Urban walls, although not conventionally considered as habitats, still show heterogeneity in terms of their physical environmental features, including substrate (stone or brick), the micro-scale profile of the wall surfaces (crack size, brick size, etc.), and the surrounding environment. Additionally, the studied Chinese cities are in the subtropical humid climate zone, which has rich species diversity and moist air is suitable for plants on a vertical space. Furthermore, under the current urban landscaping and greening culture, spontaneous species in parks and green space are often regarded as harmful weeds. Therefore, they are removed by high frequency maintenance. In comparison, walls as vertical space are less disturbed and thus such plants can grow.

The dominant families were Compositae and Urticaceae. This is consistent with earlier studies on spontaneous vegetation. Unlike two-dimensional land environment, more fern families, such as Dryopteridaceae, Pteridaceae, Dennstaedtiaceae, Sinopteridaceae and Aspleniaceae, inhabit walls. This finding confirms the results on the ancient stone wall of the Nanjing City. Even compared with Southern Hemisphere cities with a typical oceanic climate^23^, the warm temperate continental Chongqing has Dryopteridaceae and Aspleniaceae fern families in common. These results reflect the uniqueness of walls as potential “habitats”.

The dominant species are *Pteris vittata* (78.3%), *Pteris multifida*, (49.3%), seedlings of *Broussonetia papyrifera* (45.4%), *Cyrtomium fortune* (42.1%), and seedlings of *Ficus virens* (41.5%). Obviously, ferns predominate. Compared with two-dimensional land environment in cities at a similar latitude and similar climate, the dominant species are distinct. For example, the dominant species in Ningbo City include *Digitaria sanguinalis*, *Oxalis corniculata, Acalypha australis*, *Cynodon dactylon*, *Alternanthera philoxeroides* and *Conyza canadensis*^32^. The dominant species in Hangzhou City include *Broussonetia papyrifera*, *Koelreuteria paniculata*, *Pterocarya stenoptera*, *Ophiopogon japonicus*, *Humulus scandens* and *Hedera nepalensis*^35^. Conspicuously, spontaneous species in two-dimensional land environment are concentrated in xerophytes; some of them are invasive (e.g., *Humulus scandens*). Such species easily achieve dominance and accelerate population declines of urban biodiversity.

The dominant species on walls in Chongqing City are very similar to the walls of other cities with similar climatic conditions, such as cities in the Pearl River Delta region, where *Pteris vittata*, *Pteris multifidi* and *Broussonetia papyrifera* are dominant^36^. Likewise, adjacent Hongkong has *Broussonetia papyrifera* as the dominant species^10^. Additionally, *Broussonetia papyrifera, Pteris multifidi* and *Cyrtomium fortune* were dominant in the stone wall of Nanjing city^13^. The varied microclimate and socio-environmental factors around these sites and regions influence and differentiate species composition^37,38^. However, analogous to two-dimensional land environment, we speculate that there are also some species present because of certain traits such as seed dispersal and reproduction, can be distributed widely on wall habitats. This speculation requires further exploration in cities with different environment. Analysis of the functional traits of these spontaneous species (e.g., life form, dispersal type, morphological type) are needed.

For all species found in each city, the spontaneous species on walls in Chongqing have little resemblance to those on the stone wall of Nanjing city^13^, with less than 25% in common. This may be related to the fact that the local “seed bank” varies in these cities. For example, *Ficus virens* is a well-known civic tree in Chongqing and is widely cultivated in almost all types of green space. This tree belongs to the Moraceae, genus Ficus, which are generally hardy, vigorous and prolific in growth and regeneration, relying on seeds and propagules such as cuttings. Their fruits, figs, are favored by a wide range of frugivores that are capable of dispersing the seeds. Unsurprisingly, it can survive and settle in barren walls and grow into a dominant species. However, *Ficus virens* is not reported in cities at a similar latitude such as Nangjing City^13^ and Jinzhou City^39^. Likewise, *Pteris vittata* is also a common species in greening and landscaping in Chongqing. Therefore, there is a rich local “seed bank”. Plants can easily “escape” from urban green space and “spill over” into the vertical space by spores or ramets. This finding of walls having similar species to the surrounding environment is consistent with other studies^7^. This finding is incompatible with the “Urban Cliff Hypothesis”. Instead, our results in Chongqing are inclined to represent the mass effect, whereby species establish in some unsuitable habitats simply because of the abundance of propagules in the surrounding environment that ensures that some individuals establish successfully^40^. With walls, because species abundance is greatly different between urban green space (squeezed with species) and bare walls, species migrate from source to sink habitats.

### Influences on spontaneous species on walls

This study involved two types of influence and 13 indicators. However, the explanatory power of all four wall characteristics was low, less than 30%. The unexplained residual may come from aspects of the complexity of Nature. Plant species’ introduction, settling and community assembly are complex processes. On one side, introduction is affected by the species’ characteristics (such as species’ adaptation to their environment) and stochastic mechanisms across the natural environment. On the other side, the process is affected by the wall’s characteristics and the surrounding land use. Studies support the importance of land use on a large scale to species richness on a local scale^41,42^. Specifically, the study of the stone wall of Nanjing city confirmed that sampled walls located among hills, botanical gardens and urban green space tended to produce high species richness and abundance because of nearby seed sources. Although we have considered adjacent influences in this study, only the human disturbance degree and land use type were selected to measure this. More factors measuring adjacent land use and landscape patterns are lacking, such as metrics describing the composition and configuration of the surrounding landscape. Further empirical studies are therefore required to understand the coupling mechanism of wall attributes and surrounding land use on wall vegetation patterns.

As expected, wall attributes contribute the largest explanation. Among all variables, vine coverage, wall shade and wall height were the most important factors influencing vegetation composition and distribution. Vine coverage and wall shade are directly related to water availability. Rainfall is a direct source of water for plant growth. According to our observations, making full use of moisture from the air and wall is more important for spontaneous species on walls. Therefore, some plants were found having aerial roots, e.g., *Ficus virens* and other Ficus species. Subtropical cities generally have high relative humidity, like Chongqing whose annual average relative humidity is 70–80%. This allows plants to absorb adequate water from the air. However, summers are scorching and solar radiation is high (Chongqing is well-known as one of China’s “Four Big Furnaces”). Especially in today’s context of extreme weather events associated with global climate change, creating a damp, humid environment is a key for plant species’ settlement.

As a mountain city short of construction land, Chongqing has a higher density of buildings than plains cities. No matter the wall aspect, the lower part of walls is not easily exposed to sunlight and generally grows more species, while the higher wall part has less shading and may be exposed to cold winds, so it is harsh area for plant species to settle. This finding is consistent with results from a study of the ancient walls of Jingzhou City, where shadowed sides of walls had more species than the sunny side^39^. In highly dense cities, the wall aspect cannot determine sun exposure, instead wall height, to some extent, represents sun availability. Therefore, variable wall aspects showed no influence on wall species, which agrees with a study in Hongkong^7^.

Wall is a novel yet undefined habitat, where nutrients that plants require almost all come from the external environment. Therefore, the plants are greatly disturbed by externalities and often have a typical randomness and stochasticity. Compared with general urban two-dimensional land environment, the composition and distribution of spontaneous species on walls represents high uncertainty and high species turnover rate and, presumably, the influences are varied. Therefore, a long-term, tracking study over different seasons is needed.

## Conclusions and implications for current urban greening

China and some other ancient countries have a long history of building walls, from the magnificent Great Wall to local courtyard walls around houses. Even today, a growing number of walls of buildings, boundary walls and retaining walls within cities play an important demarcation and safeguarding role. The increasing amount of vertical space also opens up new opportunities for urban greening and landscaping.

This study uses a typical mountainous city, Chongqing, as an example to explore the potential of ordinary urban walls as urban habitats. A total of 239 vascular plant species belonging to 172 genera and 75 families were found. The wall habitats perform well compared with the general urban environment. Of the species, 90% are indigenous and can provide multiple ecosystem services. For example, *Ficus tikoua*, and *Setaira viridis* can provide bird and small animal food sources and stepping-stones on their migratory way, which further enhances urban biodiversity. Additionally, there are some species with higher aesthetic values, such as diverse ferns: *Fallopia multiflora* and *Paederia scandens* can be used for leaf appreciation; *Lonicera japonica* for flower appreciation; *Setaira viridis*, *Artemisia annua*, *Lagopsis supina* and *Clinopodium chinense* present another different view of wild nature in cities. Recently, greening walls studies have shifted to a growing concern over the “living wall”^43^. Compared with conventionally using cultivated climbing species for walls, living walls emphasises applying diverse indigenous species to form an assemblage and further attract small animals to cities. The spontaneous species found on walls can provide plenty of appropriate species for vertical spaces (walls, slopes, overpasses, balconies, etc.) and add a more vibrant wild atmosphere to urban landscapes.

It is worthwhile pointing out that this study does not widely encourage all buildings to be covered by spontaneous plants. Woody species may damage walls because of their weight and deep strong roots^44^. Meanwhile, we also found that some species are more likely to become invasive. Instead, we focus more on promoting alternative plant selection from such exposed and infertile habitats. Urban greening managers should change their view of and attitude towards these uncultivated species; not all are destructive weeds that need eradication. Therefore, urban managers should recognise plant species’ traits (such as the variety of life forms, native or exotic) and further formulate meticulous management strategies that could well target all kinds of circumstances, instead of a “one size fits all” approach. This could accommodate more wild indigenous species in urban environment. By making full use of the spontaneity of plant species to generate low maintenance landscapes, Nature is allowed to take its course. Confronting these unconventional, informal habitats, more empirical studies under a broader context of seasons, climate conditions and regions are required to explore cost-effective, diverse, functional, and requiring minimal maintenance landscape approaches to improve urban ecosystems.

## Supplementary information

Supplementary Information.

Supplementary Information.

Supplementary Information.

Supplementary Information.
