# Supplementary material for: Walls offer potential to improve urban biodiversity
Source: Sci Rep. 2020 Jun 18;10:9905. doi: 10.1038/s41598-020-66527-3 (PMC7303168; doi:10.1038/s41598-020-66527-3)
Supplement: Supplementary file 1 — Supplementary Information. [file 41598_2020_66527_MOESM1_ESM.docx]

All species surveyed in our study

| **Species Name** | **Family Name** | **Genus Name** |
| --- | --- | --- |
| *Pteris vittata* | Pteridaceae | Pteris |
| *Cyrtomium fortunei* | Dryopteridaceae | Cyrtomium Presl |
| *Pteris multifida* | Pteridaceae | Pteris |
| *Pteris cretica* | Pteridaceae | Pteris |
| *Youngia japonica* | Compositae | Youngia Cass. |
| *Youngia erythrocarpa* | Compositae | Youngia Cass. |
| *Ixeris polycephala* | Compositae | Ixeris Cass. |
| *Ixeridium sonchifolium* | Compositae | Ixeridium (A. Gray) Tzvel. |
| *Sonchus oleraceus* | Compositae | Sonchus L. |
| *Sambucus williamsii* | Caprifoliaceae | Sambucus Linn. |
| *Erigeron annuus* | Compositae | Erigeron L. |
| *Conyza canadensis* | Compositae | Conyza Less. |
| *Ficus virens* | Moraceae | Ficus Linn. |
| *Broussonetia papyrifera* | Moraceae | Broussonetia L’Hert. ex Vent. |
| *Ficus microcarpa* | Moraceae | Ficus Linn. |
| *Rorippa indica* | Cruciferae | Rorippa Scop. |
| *Phytolacca americana* | Phytolaccaceae | Phytolacca L. |
| *Oxalis corniculata* | Oxalidaceae | Oxalis L. |
| *Oxalis corymbosa* | Oxalidaceae | Oxalis L. |
| *Asplenium incisum* | Aspleniaceae | Asplenium L. |
| *Microsorum fortunei* | Polypodiaceae | Microsorum Link |
| *Asplenium pekinense* | Aspleniaceae | Asplenium L. |
| *Lygodium japonicum* | Lygodiaceae | Lygodium Sw. |
| *Cyclosorus parasiticus* | Thelypteridaceae | Cyclosorus Link |
| *Cyclosorus acuminatus* | Thelypteridaceae | Cyclosorus Link |
| *Achyranthes bidentata* | Amaranthaceae | Achyranthes L. |
| *Achyranthes aspera* | Amaranthaceae | Achyranthes L. |
| *Humulus scandens* | Moraceae | Humulus Linn. |
| *Poa annua* | Gramineae | Poa L. |
| *Digitaria sanguinalis* | Gramineae | Digitaria Hall. |
| *Oplismenus undulatifolius* | Gramineae | Oplismenus Beauv. |
| *Cyperus rotundus L.* | Cyperaceae | Cyperus Linn. |
| *Arthraxon hispidus* | Gramineae | Arthraxon Beauv. |
| *Acalypha australis* | Euphorbiaceae | Acalypha L. |
| *Carpesium cernuum* | Compositae | Carpesium L. |
| *Hydrocotyle sibthorpioides* | Umbelliferae | Hydrocotyle L. |
| *Pouzolzia zeylanica* | Urticaceae | Pouzolzia Gaudich. |
| *Corydalis edulis* | Papaveraceae | Corydalis DC. |
| *Nanocnide lobata* | Urticaceae | Nanocnide Bl. |
| *Nanocnide japonica* | Urticaceae | Nanocnide Bl. |
| *Ampelopsis delavayana* | Vitaceae | Ampelopsis Michaux |
| *Ampelopsis bodinieri* | Vitaceae | Ampelopsis Michaux |
| *Solanum lyratum* | Solanaceae | Solanum L. |
| *Solanum photeinocarpum* | Solanaceae | Solanum L. |
| *Solanum nigrum* | Solanaceae | Solanum L. |
| *Clinopodium chinense* | Labiatae | Clinopodium Linn. |
| *Clinopodium gracile* | Labiatae | Clinopodium Linn. |
| *Euphorbia maculata* | Euphorbiaceae | Euphorbia Linn. |
| *Parthenocissus semicordata* | Vitaceae | Parthenocissus Planch. |
| *Fallopia multiflora* | Polygonaceae | Fallopia Adans. |
| *Sinosenecio oldhamianus* | Compositae | Sinosenecio B. Nord. |
| *Aleuritopteris argentea* | Sinopteridaceae | Aleuritopteris Fee |
| *Bischofia javanica* | Euphorbiaceae | Bischofia Bl. |
| *Koelreuteria bipinnata* | Sapindaceae | Koelreuteria Laxm. |
| *Chenopodium album* | Chenopodiaceae | Chenopodium Linn. |
| *Buddleja lindleyana* | Loganiaceae | Buddleja (Buddleia auct.) Linn. |
| *Pteridium aquilinum* | Pteridiaceae | Pteridium Scopoli |
| *Adiantum capillus-veneris* | Adiantaceae | Adiantum L. |
| *Sedum lineare* | Crassulaceae | Sedum L. |
| *Polygonum chinense* | Polygonaceae | Polygonum L. |
| *Sedum emarginatum* | Crassulaceae | Sedum L. |
| *Viola inconspicua* | Violaceae | Viola L. |
| *Talinum paniculatum* | Portulacaceae | Talinum Adans. |
| *Cardamine hirsuta* | Cruciferae | Cardamine L. |
| *Cardamine griffithii* | Cruciferae | Cardamine L. |
| *Alternanthera philoxeroides* | Amaranthaceae | Alternanthera Forsk. |
| *Artemisia argyi* | Compositae | Artemisia Linn. Sensu stricto, excl. Sect. Seriphidium Bess. |
| *Crassocephalum crepidioides* | Compositae | Crassocephalum Moench |
| *Alternanthera sessilis* | Amaranthaceae | Alternanthera Forsk. |
| *Boehmeria silvestrii* | Urticaceae | Boehmeria Jacq. |
| *Alocasia macrorrhiza* | Araceae | Alocasia (Schott) G. Don |
| *Punica granatum* | Punicaceae | Punica Linn. |
| *Zehneria japonica* | Cucurbitaceae | Zehneria |
| *Anredera cordifolia* | Basellaceae | Anredera Juss. |
| *Basella alba* | Basellaceae | Basella L. |
| *Asparagus cochinchinensis* | Liliaceae | Asparagus L. |
| *Cinnamomum japonicum* | Lauraceae | Cinnamomum Trew |
| *Onychium japonicum* | Sinopteridaceae | Onychium Kaulf. |
| *Stenoloma chusanum* | Lindsaeaceae | Stenoloma Fee (Lindsaeaceae) |
| *Stellaria media* | Caryophyllaceae | Stellaria L. |
| *Phyllanthus urinaria* | Euphorbiaceae | Phyllanthus Linn. |
| *Rostellularia procumbens* | Acanthaceae | Rostellularia Reichenb. |
| *Bidens pilosa L. var. radiata* | Compositae | Bidens L. |
| *Paederia scandens* | Rubiaceae | Paederia Linn. nom. cons. |
| *Woodsia polystichoides* | Woodsiaceae | Woodsia R. Br. |
| *Dryopteris sparsa* | Dryopteridaceae | Dryopteris Adanson |
| *Microlepia strigosa* | Dennstaedtiaceae | Microlepia Presl |
| *Pilea microphylla* | Urticaceae | Pilea Lindl. |
| *Sedum filipes* | Crassulaceae | Sedum L. |
| *Ulmus pumila* | Ulmaceae | Ulmus L. |
| *Chenopodium ambrosioides* | Chenopodiaceae | Chenopodium Linn. |
| *Rubus coreanus* | Rosaceae | Rubus L. |
| *Amygdalus persica* | Rosaceae | Amygdalus L. |
| *Cynodon dactylon* | Gramineae | Cynodon Rich. |
| *Debregeasia orientalis* | Urticaceae | Debregeasia Gaudich. |
| *Eriobotrya japonica* | Rosaceae | Eriobotrya Lindl. |
| *Commelina communis* | Commelinaceae | Commelina Linn. |
| *Malvaviscus arboreus Cav. var. penduliflorus* | Malvaceae | Malvaviscus Dill. ex Adans. |
| *Mirabilis jalapa* | Nyctaginaceae | Mirabilis L. |
| *Lonicera japonica* | Caprifoliaceae | Lonicera Linn. |
| *Pilea japonica* | Urticaceae | Pilea Lindl. |
| *Reineckia carnea* | Liliaceae | Reineckia Kunth |
| *Cayratia japonica* | Vitaceae | Cayratia Juss. |
| *Campsis grandiflora* | Bignoniaceae | Campsis Lour. |
| *Rhododendron simsii* | Ericaceae | Rhododendron L. |
| *Duchesnea indica* | Rosaceae | Duchesnea J. E. Smith |
| *Blumea aromatica* | Compositae | Blumea DC. |
| *Ophiopogon japonicus* | Liliaceae | Ophiopogon Ker-Gawl. |
| *Plantago depressa* | Plantaginaceae | Plantago L. |
| *Carex baccans* | Cyperaceae | Carex Linn. |
| *Lindera megaphylla* | Lauraceae | Lindera Thunb. |
| *Cinnamomum camphora* | Lauraceae | Cinnamomum Trew |
| *Cyclobalanopsis glauca* | Fagaceae | Cyclobalanopsis Oerst. |
| *Microlepia marginata* | Dennstaedtiaceae | Microlepia Presl |
| *Myosoton aquaticum* | Caryophyllaceae | Myosoton Moench |
| *Alangium chinense* | Alangiaceae | Alangium Lam. |
| *Aralia chinensis* | Araliaceae | Aralia Linn. |
| *Setaria viridis* | Gramineae | Setaria Beauv. |
| *Corydalis f. bulbillifera* | Papaveraceae | Corydalis DC. |
| *Centella asiatica* | Umbelliferae | Centella L. |
| *Ficus tikoua* | Moraceae | Ficus Linn. |
| *Setaria plicata* | Gramineae | Setaria Beauv. |
| *Setaria palmifolia* | Gramineae | Setaria Beauv. |
| *Mahonia fortunei* | Berberidaceae | Mahonia Nuttall |
| *Celtis biondii* | Ulmaceae | Celtis L. |
| *Celtis sinensis* | Ulmaceae | Celtis L. |
| *Polystichum tripteron* | Dryopteridaceae | Polystichum Roth |
| *Nephrolepis auriculata* | Nephrolepidaceae | Nephrolepis Schott |
| *Drynaria roosii* | Drynariaceae | Drynaria (Bory) J. Sm. |
| *Zanthoxylum armatum* | Rutaceae | Zanthoxylum L. |
| *Lysimachia christinae* | Primulaceae | Lysimachia L. |
| *Selaginella uncinata* | Selaginellaceae | Selaginella P. Beauv. |
| *Dichondra repens* | Convolvulaceae | Dichondra J. R. et G. Forst. |
| *Padus buergeriana* | Rosaceae | Padus Mill. |
| *Veronica undulata* | Scrophulariaceae | Veronica L. |
| *Sagina japonica* | Caryophyllaceae | Sagina L. |
| *Hylocereus undatus* | Cactaceae | Hylocereus (Berg.) Britt. et Rose |
| *Eclipta prostrata* | Compositae | Eclipta L. |
| *Quercus phillyraeoides* | Fagaceae | Quercus L. |
|  |  |  |
| *Girardinia suborbiculata subsp. triloba* | Urticaceae | Girardinia Gaudich. |
| *Viola diffusa* | Violaceae | Viola L. |
| *Pyracantha fortuneana* | Rosaceae | Pyracantha Roem. |
| *Typhonium divaricatum* | Araceae | Typhonium Schott |
| *Gynostemma pentaphyllum* | Cucurbitaceae | Gynostemma Bl. |
| *Artemisia lactiflora* | Compositae | Artemisia Linn. Sensu stricto, excl. Sect. Seriphidium Bess. |
| *Acanthopanax trifoliatus* | Araliaceae | Acanthopanax Miq. |
| *Citrus maxima* | Rutaceae | Citrus L. |
| *Boehmeria nivea* | Urticaceae | Boehmeria Jacq. |
| *Lycium chinense* | Solanaceae | Lycium L. |
| *Carpesium abrotanoides* | Compositae | Carpesium L. |
| *Oreocnide frutescens* | Urticaceae | Oreocnide Miq. |
| *Ficus pandurata* | Moraceae | Ficus Linn. |
| *Viola philippica* | Violaceae | Viola L. |
| *Galium aparine Linn. var. echinospermum* | Rubiaceae | Galium Linn. |
| *Lagopsis supina* | Labiatae | Lagopsis Bunge ex Benth. |
| *Mazus japonicus* | Scrophulariaceae | Mazus Lour. |
| *Echinochloa crusgalli* | Gramineae | Echinochloa Beauv. |
| *Polygonum longisetum* | Polygonaceae | Polygonum L. |
| *Pharbitis purpurea* | Convolvulaceae | Pharbitis Choisy |
| *Aster tataricus* | Compositae | Aster L. |
| *Amaranthus lividus* | Amaranthaceae | Amaranthus L. |
| *Erythrina variegata* | Leguminosae | Erythrina Linn. |
| *Dendranthema indicum* | Compositae | Dendranthema (DC.) Des Moul. |
| *Eupatorium coelestinum* | Compositae | Eupatorium L. |
| *Ailanthus altissima* | Simaroubaceae | Ailanthus Desf. |
| *Rumex acetosa* | Polygonaceae | Rumex L. |
| *Sedum stellariifolium* | Crassulaceae | Sedum L. |
| *Miscanthus sinensis* | Gramineae | Miscanthus Anderss. |
| *Akebia trifoliata* | Lardizabalaceae | Akebia Decne. |
| *Polygonum lapathifolium* | Polygonaceae | Polygonum L. |
| *Kyllinga brevifolia* | Cyperaceae | Kyllinga Rottb. |
| *Vicia sepium* | Leguminosae | Vicia Linn. |
| *Clinopodium urticifolium* | Labiatae | Clinopodium Linn. |
| *Ligustrum lucidum* | Oleaceae | Ligustrum Linn. |
| *Ajuga decumbens* | Labiatae | Ajuga Linn. |
| *Iris japonica* | Iridaceae | Iris L. |
| *Dennstaedtia pilosella* | Dennstaedtiaceae | Dennstaedtia Bernh. |
| *Dryopteris erythrosora* | Dryopteridaceae | Dryopteris Adanson |
| *Polygonum hydropiper L.* | Polygonaceae | Polygonum L. |
| *Gnaphalium affine* | Compositae | Gnaphalium L. |
| *Selaginella nipponica* | Selaginellaceae | Selaginella P. Beauv. |
| *Dichrocephala auriculata* | Compositae | Dichrocephala DC. |
| *Cheilosoria mysurensis* | Sinopteridaceae | Cheilosoria Trev. |
| *Robinia pseudoacacia* | Leguminosae | Robinia Linn. |
| *Chlorophytum comosum* | Liliaceae | Chlorophytum Ker-Gawl. |
| *Senecio scandens* | Compositae | Senecio L. |
| *Saxifraga stolonifera* | Saxifragaceae | Saxifraga Tourn. ex L. |
| *Boehmeria densiglomerata* | Urticaceae | Boehmeria Jacq. |
| *Artemisia japonica* | Compositae | Artemisia Linn. Sensu stricto, excl. Sect. Seriphidium Bess. |
| *Gynura bicolor (Roxb. ex Willd.) DC.* | Compositae | Gynura Cass. nom. cons. |
| *Allium tuberosum* | Liliaceae | Allium L. |
| *Hibiscus trionum* | Malvaceae | Hibiscus Linn. |
| *Plantago virginica* | Plantaginaceae | Plantago L. |
| *Pterocarya stenoptera* | Juglandaceae | Pterocarya Kunth |
| *Pistacia chinensis* | Anacardiaceae | Pistacia L. |
| *Morus alba* | Moraceae | Morus Linn. |
| *Sophora japonica* | Leguminosae | Sophora Linn. |
| *Melia azedarach* | Meliaceae | Melia Linn. |
| *Sapium sebiferum* | Euphorbiaceae | Sapium P. Br. |
| *Parathelypteris glanduligera* | Thelypteridaceae | Parathelypteris (H. Ito) Ching |
| *Asplenium trichomanes* | Aspleniaceae | Asplenium L. |
| *Pyrrosia petiolosa* | Polypodiaceae | Pyrrosia Mirbel |
| *Pteris actiniopteroides* | Pteridaceae | Pteris L. |
| *Lindernia ruellioides* | Scrophulariaceae | Lindernia All. |
| *Allium chrysanthum* | Liliaceae | Scrophulariaceae |
| *Jasminum mesnyi* | Oleaceae | Jasminum Linn. |
| *Selaginella moellendorffii* | Selaginellaceae | Selaginella P. Beauv. |
| *Eragrostis minor* | Gramineae | Eragrostis Wolf |
| *Euphorbia humifusa* | Euphorbiaceae | Euphorbia Linn. |
| *Houttuynia cordata* | Saururaceae | Houttuynia Thunb. |
| *Lysimachia congestiflora* | Primulaceae | Lysimachia L. |
| *Pilea notata* | Urticaceae | Pilea Lindl. |
| *Clinopodium confine* | Labiatae | Clinopodium Linn. |
| *Ficus gasparriniana Miq. var. laceratifolia* | Moraceae | Ficus Linn. |
| *Verbena officinalis* | Verbenaceae | Verbena Linn. |
| *Lindernia crustacea* | Scrophulariaceae | Lindernia All. |
| *Veronica didyma* | Scrophulariaceae | Veronica L. |
| *Conyza bonariensis* | Compositae | Conyza Less. |
| *Calystegia hederacea* | Convolvulaceae | Calystegia R. Br. |
| *Boehmeria clidemioides* | Urticaceae | Boehmeria Jacq. |
| *Youngia heterophylla* | Compositae | Youngia Cass. |
| *Leonurus artemisia* | Labiatae | Leonurus Linn. |
| *Iris tectorum* | Iridaceae | Iris L. |
| *Rumex crispus* | Polygonaceae | Rumex L. |
| *Mucuna sempervirens* | Leguminosae | Mucuna Adans. |
| *Sedum sarmentosum* | Crassulaceae | Sedum L. |
| *Celastrus gemmatus* | Celastraceae | Celastrus L. |
| *Chrysosplenium macrophyllum* | Saxifragaceae | Chrysosplenium Tourn. ex L. |
| *Tradescantia zebrina* | Commelinaceae | Tradescantia |
| *Glechoma longituba* | Labiatae | Glechoma Linn. |
| *Paulownia fortunei* | Scrophulariaceae | Paulownia Sieb. et Zucc. |
| *Trifolium repens* | Leguminosae | Trifolium Linn. |
| *Liriope spicata* | Liliaceae | Liriope Lour. |
| *Phytolacca acinosa* | Phytolaccaceae | Phytolacca L. |
| *Broussonetia kaempferi Sieb. var. australis* | Moraceae | Broussonetia L’Hert. ex Vent. |
| *Physalis alkekengi* | Solanaceae | Physalis L. |
| *Perilla frutescens* | Labiatae | Perilla Linn. |
| *Setcreasea purpurea* | Commelinaceae | Commelina Linn. |
| *Commelina purpurea* | Commelinaceae | Commelina Linn. |
