# Supplementary material for: Walls offer potential to improve urban biodiversity
Source: Sci Rep. 2020 Jun 18;10:9905. doi: 10.1038/s41598-020-66527-3 (PMC7303168; doi:10.1038/s41598-020-66527-3)
Supplement: Supplementary file 2 — Supplementary Information. [file 41598_2020_66527_MOESM2_ESM.docx]

**ANOVA comparison of plant species richness among three land use/cover types (parks, abandoned areas and concrete environment)**

| **ANOVA** | | | | | |
| --- | --- | --- | --- | --- | --- |
| number | | | | | |
|  | Sum of Squares | df | Mean Square | F | Sig. |
| Between Groups | 156.755 | 1 | 156.755 | 10.937 | .003 |
| Within Groups | 300.984 | 21 | 14.333 |  |  |
| Total | 457.739 | 22 |  |  |  |

**Analysis 'Interactive-forward-selection', step 'Forward Selection'**

Method: CCA

Total variation is 12.42098, explanatory variables account for 43.0%

(adjusted explained variation is 28.3%)

Summary Table:

Statistic Axis 1 Axis 2 Axis 3 Axis 4

Eigenvalues 0.7382 0.5952 0.4298 0.3485

Explained variation (cumulative) 5.94 10.73 14.19 17.00

Pseudo-canonical correlation 0.9418 0.9036 0.8378 0.8162

Explained fitted variation (cumulative) 21.19 38.27 50.61 60.61

Analysis 'Interactive-forward-selection', step 'Forward Selection'

Forward Selection Results:

Name Explains % Contribution % pseudo-F P

Vicov 5.2 14.2 4.9 0.002

Sh 4.8 13.4 4.7 0.002

Hi 4.5 12.4 4.4 0.002

We 3.4 8.6 3.1 0.004

Man 3.2 7.1 3.0 0.002

LuLc 3.0 6.6 2.4 0.006

Co 2.7 5.7 2.1 0.002

JoMa 2.6 5.2 2.0 0.008

Hu 2.5 5.0 1.9 0.004

SI 2.4 4.8 1.8 0.004

Di 2.4 5.1 2.0 0.004

Joden 2.3 5.8 2.7 0.002

Josi 2.1 3.7 1.6 0.024

Ma 1.9 2.3 1.2 0.025

======================
