# Supplementary material for: Walls offer potential to improve urban biodiversity
Source: Sci Rep. 2020 Jun 18;10:9905. doi: 10.1038/s41598-020-66527-3 (PMC7303168; doi:10.1038/s41598-020-66527-3)
Supplement: Supplementary file 3 — Supplementary Information. [file 41598_2020_66527_MOESM3_ESM.docx]

**Variation partitioning for the two groups of the wall attributes and adjacent influences**

Variation Partitioning Results for Two Groups in Analysis 'Var-part-2groups-Conditional-effects-tested'

Explained variation

Fraction Variation(adj) % of Explained % of All DF Mean Square

a 1.5353 77.1 13.6 11 0.17962

b 1.07388 11.5 9.5 4 0.15647

c 0.7014 11.3 6.2 2 0.09315

Total Explained 3.3106 100.0 29.3 17 0.19083

All Variation 11.281 -- 100.0 119 --

Variation Partitioning Results for Two Groups in Analysis 'Var-part-2groups-Conditional-effects-tested'

Significance tests

Tested Fraction F P

a+b+c 2.3 0.002

a 2.2 0.002

b 1.9 0.002

c 1.0 0.002

**Variation partitioning for the three subgroup within the wall attributes group**

Variation Partitioning Results for Three Groups in Analysis 'Var-part-3groups-Conditional-effects-tested'

Explained variation

Fraction Variation(adj) % of Explained % of All DF Mean Square

a 0.51892 38.8 4.6 5 0.17583

b 0.39483 27.0 3.5 3 0.18863

c 0.23690 11.5 2.1 3 0.12873

d 0.02256 8.4 0.2 -- --

e 0.01128 2.0 0.1 -- --

f 0.03384 6.6 0.3 -- --

g 0.02256 5.6 0.2 -- --

Total Explained 1.2396 100.0 11.0 11 0.19708

All Variation 11.281 -- 100.0 119 --

Variation Partitioning Results for Three Groups in Analysis 'Var-part-3groups-Conditional-effects-tested'

Significance tests

Tested Fraction F P

a+b+c+d+e+f+g 2.3 0.002

a 2.1 0.002

b 2.2 0.002

c 1.5 0.002

Variation Partitioning Results for Three Groups in Analysis 'Var-part-3groups-Conditional-effects-tested'

Group Members:

First Group Second Group Third Group

Hu Sl CaMa

Wicov Co Casi

Sh Hi Caden

Ma

We
