# Supplementary material for: Walls offer potential to improve urban biodiversity
Source: Sci Rep. 2020 Jun 18;10:9905. doi: 10.1038/s41598-020-66527-3 (PMC7303168; doi:10.1038/s41598-020-66527-3)
Supplement: Supplementary file 4 — Supplementary Information. [file 41598_2020_66527_MOESM4_ESM.docx]

**Walls offer potential to improve urban biodiversity**

CHEN Chundi^1†^, MAO Longfei^2†*^, QIU Yonggui^1^, CUI Jian^3^, WANG Yuncai^1*^

1. College of Architecture and Urban Planning, Tongji University, Shanghai, China

2. College of Biology, Hunan University, Hunan, China

3. Institute of Botany, Jiangsu Province and Chinese Academy of Sciences

**First authors:**

CHEN Chundi

College of Architecture and Urban Planning, Tongji University, 1239 Siping Road, Shanghai, 200092, China.

[chundichen@tongji.edu.cn](mailto:chundichen@tongji.edu.cn); [chundi.chen@gmail.com](mailto:chundi.chen@gmail.com)

Mobil: (+86) 19171262850

Add: No. 1239 Siping Road, Yangpu District, Shanghai, 200092, China

MAO Longfei

College of Biology, Hunan University, Changsha, Hunan, 410082, China [longfeimao99@gmail.com](mailto:longfeimao99@gmail.com)

† Both two authors contributed equally to this work.

**Corresponding authors:**

MAO Longfei

College of Biology, Hunan University, Hunan, China [longfeimao99@gmail.com](mailto:longfeimao99@gmail.com)

WANG Yuncai

College of Architecture and Urban Planning, Tongji University, Shanghai, China

[wyc1967@tongji.edu.cn](mailto:wyc1967@tongji.edu.cn)

**Other authors:**

QIU Yonggui

College of Architecture and Urban Planning, Tongji University, Shanghai, China

[aprilQYG@outlook.com](mailto:aprilQYG@outlook.com)

CUI Jian

Institute of Botany, Jiangsu Province and Chinese Academy of Sciences, Nanjing Botanical Garden, Mem. Sun Yat-Sen, Nanjing, 210014, China

[cuijianx@163.com](mailto:cuijianx@163.com)
